# Supplementary material for: The Entire Intestinal Tract Surveillance Using Capsule Endoscopy after Immune Checkpoint Inhibitor Administration: A Prospective Observational Study
Source: Diagnostics (Basel). 2021 Mar 18;11(3):543. doi: 10.3390/diagnostics11030543 (PMC8003297; doi:10.3390/diagnostics11030543)
Supplement: Supplementary file 1 [file diagnostics-11-00543-s001.zip › Figures and Tables/Table S2.docx]

**Supplementary Table 2** Characteristics of the 7 patients who could not undergo capsule endoscopy.

| **No** | **Sex** | **Age** | **PS** | **Primary**  **tumor location** | **Treatment**  **line** | **Reasons of not performing capsule endoscopy** | **The presence of abdominal**  **pain and/or**  **use of painkiller** | **Lichtiger score** | **Confirmed irAEs, grade** |
| --- | --- | --- | --- | --- | --- | --- | --- | --- | --- |
| **1** | M | 70 | 2 | RCC | Nivolumab  3rd | Fractured femur due to fall | no | 0 | None |
| **2** | M | 68 | 1 | GC | Nivolumab  3rd | Rapid disease progression | no | 0 | None |
| **3** | M | 76 | 2 | GC | Nivolumab  3rd | Grade 2 constipation due to the peritoneum metastases | yes | 6 | None |
| **4** | M | 69 | 0 | GC | Nivolumab  3rd | Grade 2 fatigue | yes | 0 | Hypothyroidism G2 |
| **5** | M | 72 | 2 | ESCC | Nivolumab  2nd | Rapid disease progression | yes | 0 | None |
| **6** | M | 67 | 1 | GC | Nivolumab  3rd | Declined consent | yes | 0 | None |
| **7** | F | 55 | 3 | GC | Nivolumab  3rd | Rapid disease progression | yes | 0 | None |

Abbreviations: ESCC, esophageal squamous cell carcinoma; F, female; GC, gastric cancer; irAEs, immune-related adverse events; M, male; PS, performance status; RCC, renal cell carcinoma.
