# Supplementary material for: The Entire Intestinal Tract Surveillance Using Capsule Endoscopy after Immune Checkpoint Inhibitor Administration: A Prospective Observational Study
Source: Diagnostics (Basel). 2021 Mar 18;11(3):543. doi: 10.3390/diagnostics11030543 (PMC8003297; doi:10.3390/diagnostics11030543)
Supplement: Supplementary file 1 [file diagnostics-11-00543-s001.zip › Figures and Tables/Table S1.docx]

**Supplementary Table 1** Time schedule of the study.

|  |  |  |
| --- | --- | --- |
| **Day** | **Timing** | **Procedure** |
| **Previous day** | Lunch, snack, dinner | Eat a low-fiber diet |
| **Examination day** | 6:00 am | Administer 1000 mL of PEG |
|  | 9:00 am | Swallow CCE-2 with dimethicone and castor oil 30 mL |
|  | 10:00 am | Confirm CCE-2 in the small intestine  Add 500 mL of PEG within 30 min and castor oil 30 mL after confirmation of CCE-2 in the small intestine |
|  | 12:00 am | Add 500 mL of PEG |
|  | 2:00 pm | Add 500 mL of water |
|  | 4:00 pm | Allowed to eat food |
| PEG, polyethylene glycol solution; CCE-2, second-generation colon capsule endoscopy | | |
