# Supplementary figures and images for: The Entire Intestinal Tract Surveillance Using Capsule Endoscopy after Immune Checkpoint Inhibitor Administration: A Prospective Observational Study
Source: Diagnostics (Basel). 2021 Mar 18;11(3):543. doi: 10.3390/diagnostics11030543 (PMC8003297; doi:10.3390/diagnostics11030543)

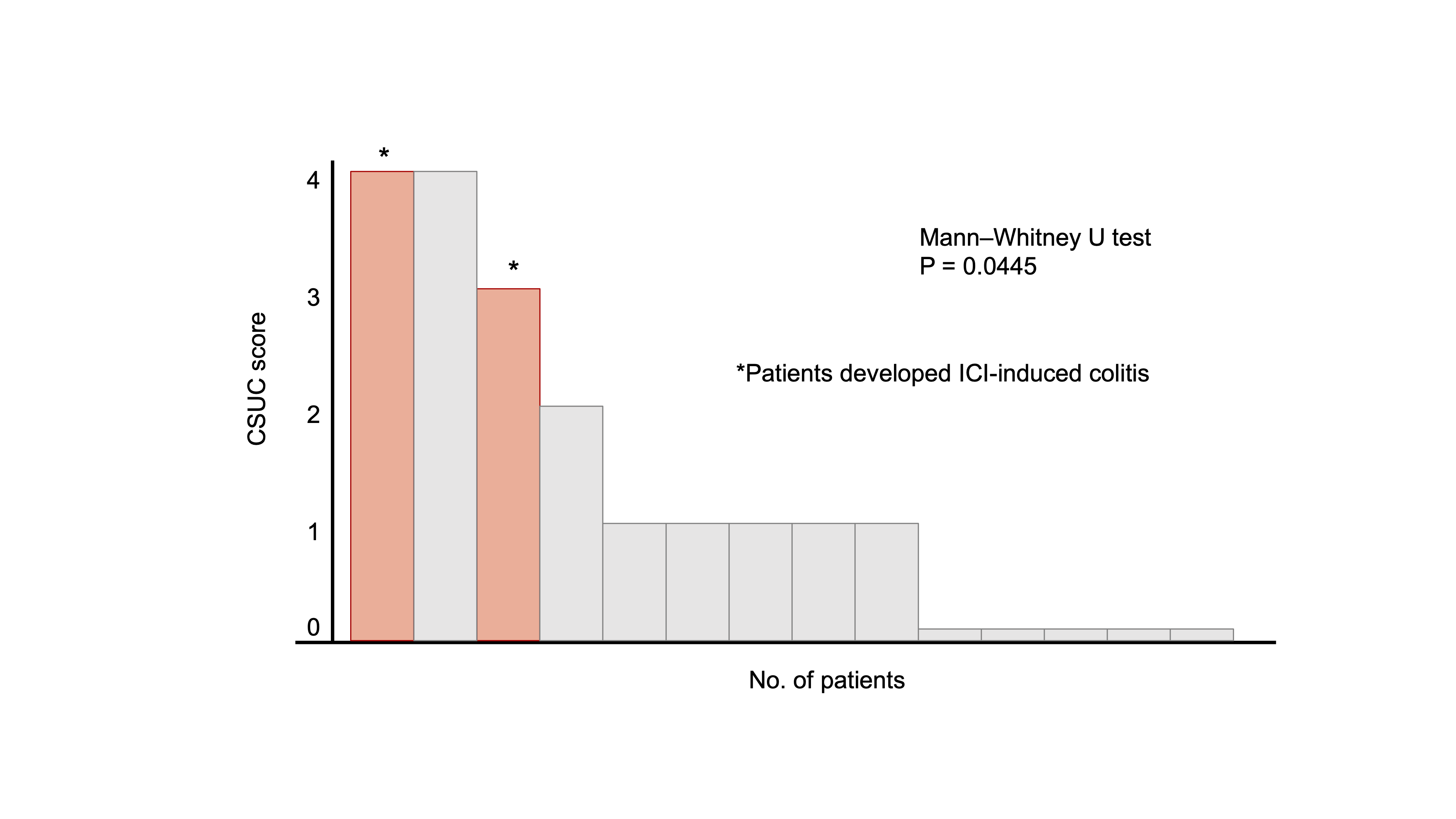

Supplement: Supplementary file 1 [file diagnostics-11-00543-s001.zip › Figures and Tables/Figure 4.tiff]

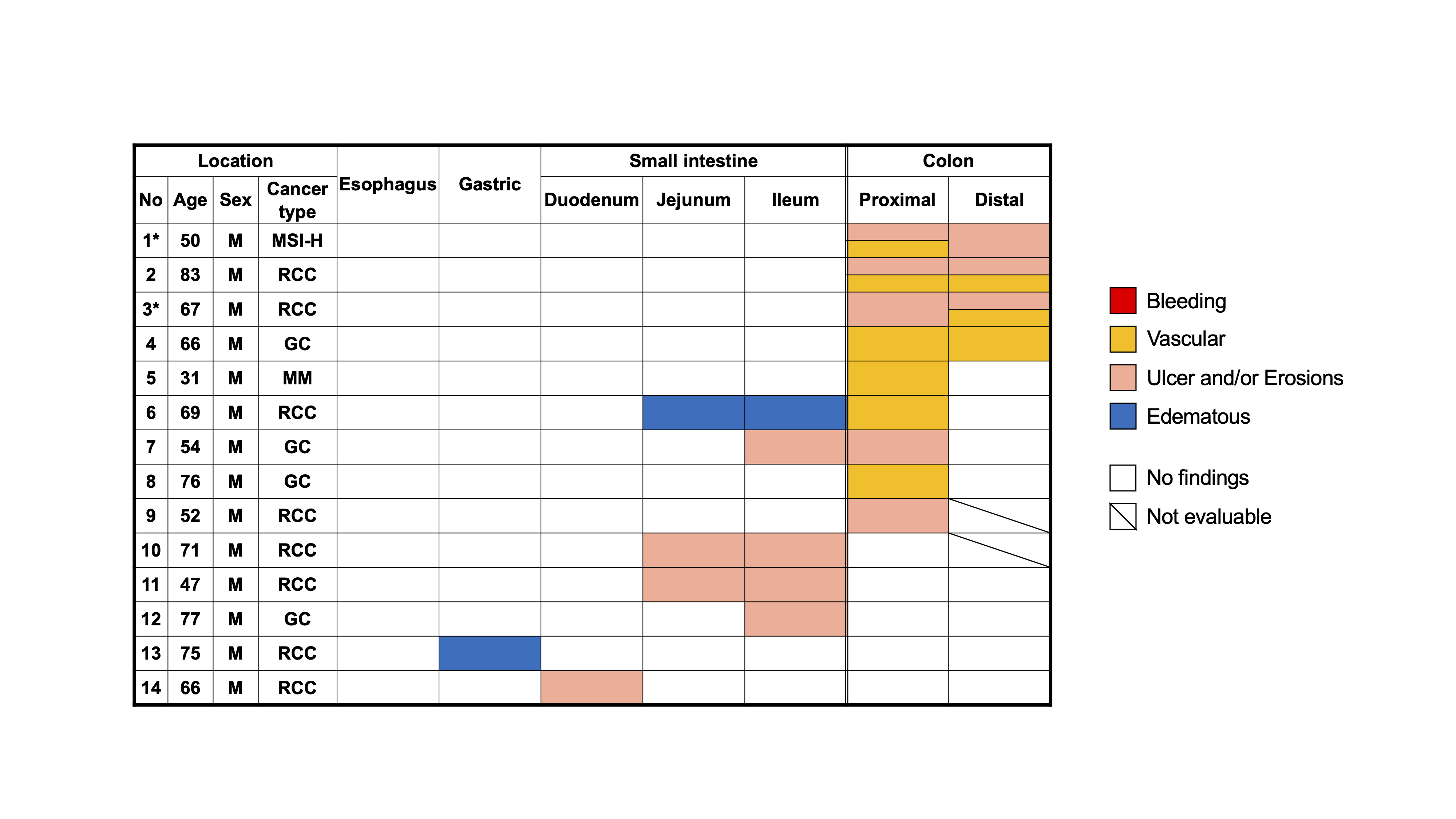

Supplement: Supplementary file 1 [file diagnostics-11-00543-s001.zip › Figures and Tables/Figure 2.tiff]

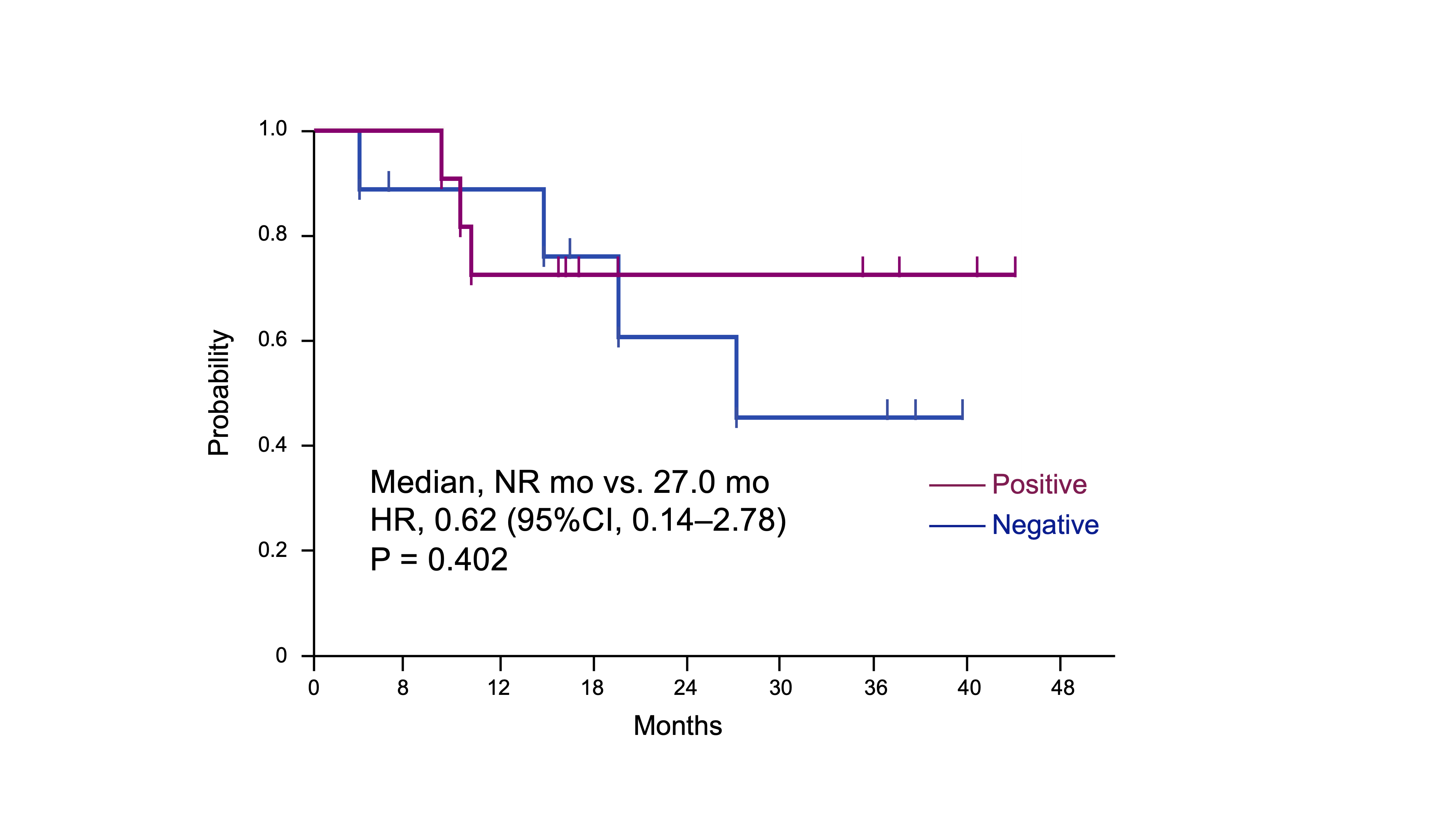

Supplement: Supplementary file 1 [file diagnostics-11-00543-s001.zip › Figures and Tables/Supplementary Figure 2.tiff]

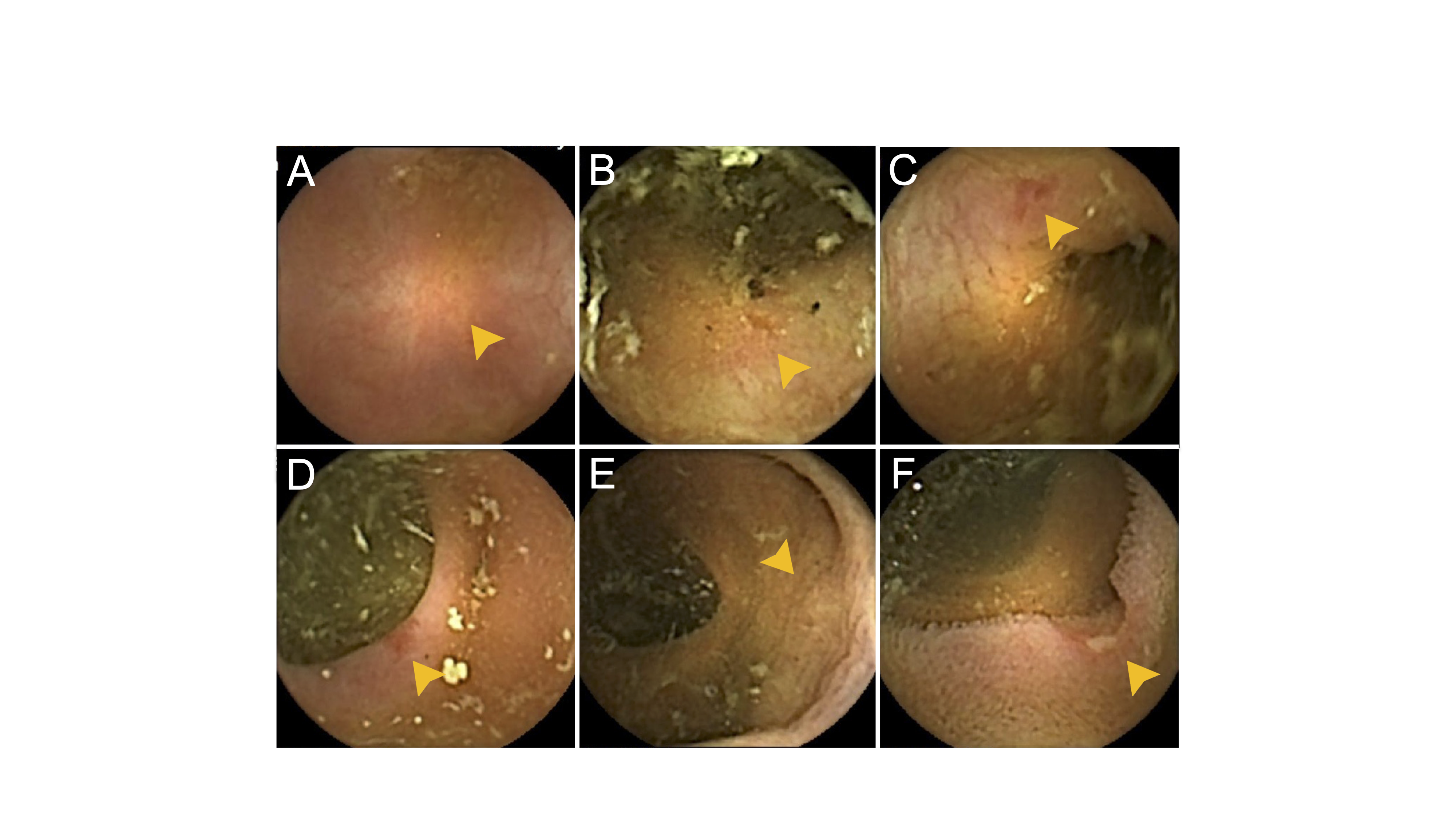

Supplement: Supplementary file 1 [file diagnostics-11-00543-s001.zip › Figures and Tables/Figure 3.tiff]

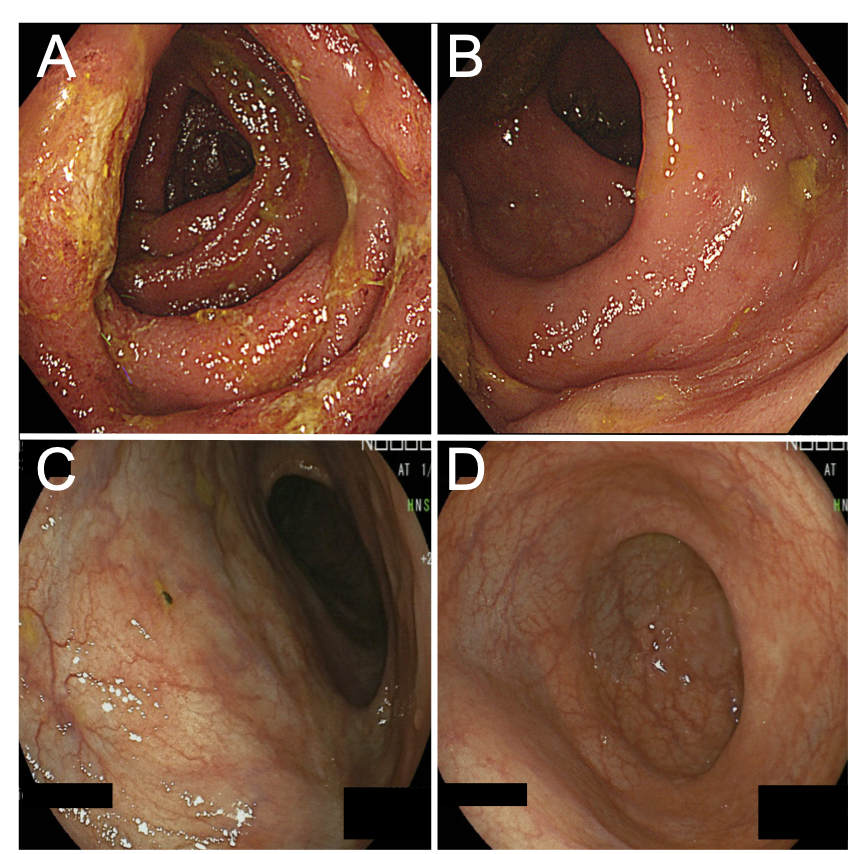

Supplement: Supplementary file 1 [file diagnostics-11-00543-s001.zip › Figures and Tables/Supplementary Figure 1.tiff]
